# Influence of Support for Career Development Intelligence Building for College Graduates on their Job Performance: The Case of South Korea

**DOI:** 10.3390/bs12090321

**Published:** 2022-09-05

**Authors:** Eun Bee Kim, Jhong Yun (Joy) Kim

**Affiliations:** 1 Department of Liberal Arts, Wesley Creative Convergence College, HyupSung University, Hwaseong-si 18330, Korea; 2 BK21 FOUR R&E Center for Education, Korea University, Seoul 02841, Korea

**Keywords:** college graduates, early-career employee, support for career development intelligence building, learning transfer, self-esteem, job performance

## Abstract

This study conducted to identify the influence of support for career development intelligence building for college graduates, who are early career employees, and to prove the mediating effect of learning transfer and self-esteem. An analysis was conducted using the Graduate Occupational Mobility Survey panel data from the Korea Employment Information Service. The data analysis was conducted using the SPSS21.0, AMOS22.0, and PROCESS macro programs. The findings are as follows. The influence of support for career development intelligence building on learning transfer is significantly positive, as is the influence of learning transfer on self-esteem. In addition, self-esteem had a significantly positive influence on job performance. However, the relationship between learning transfer and job performance was negative, and the relationship between support for career development intelligence building and self-esteem was insignificant. Lastly, the PROCESS macro analysis showed a mediating effect of learning transfer and self-esteem between support for career development intelligence building and job performance. Thus, it is suggested that support for career development intelligence building for early career employees should precede the development of specific programs to increase their self-esteem and enhance their job performance.

## 1. Introduction

Strategic human resource management is becoming a hot topic and one of the core management strategies as the competitive corporate environment intensifies, and the perception that human resources with unique valuable capabilities and technologies are sources of competitive advantage. In other words, it is changing into a strategic human resource management method that plans human resource allocation and activities to achieve organizational goals, links the management strategy process with the practice of HRD, and emphasizes the composition or coordination of various human resource management practices horizontally [1].

Career development within organizations is as an agreed strategy for mutual development between individuals and organizations by designing and managing opportunities for self-development and self-realization [2]. The knowledge obtained from support for career development intelligence building for employees helps people in their early careers perform their duties more efficiently and effectively. This improvement in performance leads to fewer mistakes, higher confidence levels, and pride in their duties [3]. Organizations need commitment and devotion from excellent talents to make it through an uncertain business environment and maintain constant growth. In response, individuals prioritize securing their employability and focus on developing themselves by going beyond the borders of organizations [4].

In exploring the effects of organizational support for career development intelli-gence building for employees on job performance, the effects of organizational invest-ment on support for career development intelligence building on the attitudes and minds of individual workers have been overlooked [5]. Theories on human resource development point out that organizational education and personal learning influence organizational performance—for instance, sales or net profit—by changing the performance and attitudes of workers, such as organizational commitment or turnover intention, rather than directly influencing organizational performance [6].

Humanware is regarded as a significant business activity factor, together with hardware and software [7]. As human resources are managers who lead management activities by utilizing material resources, the success or failure of small and medium-sized businesses depends on how human resources are managed [8]. As businesses investing heavily in human resources, evaluating the advantages of investing in career development intelligence building for small and medium-sized businesses is becoming an important activity [9].

This study aims to analyze the structural relationship between career development intelligence building, learning transfer, and self-esteem to reinforce the job performance of new employees. The results of this study will be used as empirical data to support the career development and effective human resource management of new employees. It explores the effects of support for career development intelligence building for newly hired workers in small- and medium-sized businesses on job performance. Moreover, it attempts to prove the mediating effect of self-esteem and learning transfer on the effects of support for career development intelligence building on job performance. The study aims to answer the following research questions:

**Research Question 1:** How does support for career development and intelligence building for college graduates influence job performance, learning transfer, and self-esteem?

**Research Question 2:** Do self-esteem and learning transfer mediate the relationship between support for career development intelligence building for college graduates and their job performance?

## 2. Review of Literature

### 2.1. Support for Career Development Intelligence Building

Organizational support for career development intelligence building in relation to its application to actual work is divided into training related to workers’ personal work, education related to assigned jobs that individual workers prepare, and development for individual and organizational growth. The fundamental purpose of support for intelligence building in career development can be seen as human resource development [10] or as a learning experience for improving job performance [2].

Organizational support for intelligence building in career development is divided into educational training and organizational development, depending on their purposes. Organizational support for career development intelligence building aims to foster the skills necessary for workers of performance based on various learning types. However, although this may result in better performance in a controlled learning environment in the short term, it may not bring fundamental motivation-based effects in the long term. The effects of organizational support on career development intelligence building in terms of workers and managers are as follows. For workers, organizational support for career development intelligence building provides corporate information and job knowledge, improves proficiency, reduces dissatisfaction and turnover caused by job understanding, and motivates performance goals and self-improvement. Meanwhile, for managers, the organizational support for career development intelligence building fosters talents quickly responding to rapidly changing business environments, systematizing the support for career development intelligence building based on performance evaluation, encouraging the development of personal skills based on performance evaluation, and leading personal growth to organizational growth [11]. 

### 2.2. Self-Esteem

Self-esteem shows the subjective evaluation of one’s worth and is related to the evaluation of one’s self. In other words, self-esteem is how much one appreciates oneself. People with high self-esteem are positive and satisfied with themselves, while those with low self-esteem are concerned about their weaknesses or shortcomings. Previous studies have regarded self-esteem as a self-image connected to the emotional elements of likes and dislikes [9]. In simpler terms, self-esteem is an attitude toward oneself based on self-evaluation.

According to Kahn [12], people with low self-esteem fail in their plans or dreams because they think they lack the talent to achieve their dreams or plans. On the contrary, people with higher self-esteem have more positive thoughts about their success and higher confidence in their success or achievement [5]. According to Jo et al [13], individuals with high self-esteem accept themselves just as they are and are satisfied with themselves. As people with high self-esteem believe in themselves and develop further, it is vital to strengthen their ability to trust and believe in themselves.

### 2.3. Learning Transfer

Learning transfer is an important mechanism for applying what workers learn. Kim and Han [14] defined learning transfer as effectively applying knowledge, skills, behaviors, and cognitive strategies acquired from education and learning on the job. Broad and New Ström [15] pointed out that learning transfer is defined as applying the knowledge and skills acquired from the training on the job effectively and constantly [16]. Other researchers [17,18] who studied learning transfer defined it similarly.

In previous research, learning transfer was defined as maintaining the positive changes from the acquired knowledge, skills, and attitudes on the job after completing organizational support for career development intelligence building [19] in addition to applying and maintaining the knowledge and skills acquired from the curriculum on the job [20]. Moreover, Kim, Jeon, and Chio [21] defined learning transfer as the behavior of utilizing the knowledge and skills acquired from the support for career development intelligence building on the job; Egan et al. [22] defined it as applying what one acquired from the support for career development intelligence building on the job and keeping up with the changes by emphasizing the maintenance of such skills, knowledge, and behavior after learning. Prior research has shown that learning transfer can be defined as the ability to apply what is learned from support for career development intelligence building on the job and keeping up with them.

To encourage such learning transfer, it is necessary to create a job application environment similar to the educational and training environment so that workers can actively transfer what they have learned. It is also necessary to suggest methods and places of application for the job and related procedural characteristics [5]. Specifically, the workers need to be provided with various examples for utilizing such knowledge or skills directly at work and applying them on the job immediately after the education. There also needs to be proper education methods so that learners have confidence in applying such knowledge or skills to the job.

Meanwhile, synchronization is important for smooth learning transfer in support of career development intelligence building. Kim [23] divided motivation into learning motivation and transfer motivation, pointing out that learning and transfer can ultimately happen only when the learners have transfer motivation based on the connection with the actual job and knowledge and skills they acquired. At its core, learning motivation refers to learners’ engagement in learning skills and knowledge from education and learning processes. In learning transfer, learning motivation plays a direct and continuous role in applying acquired skills and knowledge on the job more smoothly [8,13,14].

Furthermore, the importance of transfer motivation has been spotlighted recently [24,25], which indicates the engagement of learners to apply the knowledge, skills, and attitudes acquired from education to one’s job [7,26]. Holton [27] explained that transfer motivation directly influences learning transfer. Studies that covered transfer motivation as the primary variable [7,26,28,29] have shown a positive or influencing relationship between transfer motivation and learning transfer.

### 2.4. Job Performance

Job performance is defined as the results achieved by executing a task as specified [30]. In other words, job performance is the act of deriving the results required for the job [31]. In the past, financial and economic indices were mainly adopted as job performance assessment criteria in the management and human resource management fields. For instance, Coopersmith [32] defines job performance based on productivity, accident rate, and accuracy. Job performance is assessed by considering not only the economic indexes but also the behavioral aspects of the service industries [33].

This is because the business activities undertaken to fulfill business goals are also defined as job performance [34]. Job performance is evaluated using variables such as wage satisfaction, job satisfaction, and job commitment [35]. It is worth noting that research on flight attendants uses service performance to assess job performance [19,36], while research on nurses uses job performance as nurses’ professional job competence [37,38].

Apropos of viewing job performance as the result of fulfilling a duty through organizational support, providing the opportunity to develop job performance and competence, encouraging higher participation, and providing the opportunity to develop oneself have been adopted as factors for assessing job performance [39,40,41]. As previous research has shown, the definition of job performance and its measurement methods differ depending on the research targets and organizational context [42].

However, job performance needs to be regarded as the act of deriving results from behaviors rather than regarding it as simple results or behavior itself [43]. The study by Yang, Park, and Lee [44] regarded job performance as organizational behavior and pointed out that the awareness perceived by workers, motivation and efforts, as well as job competence and roles can be used as factors for identifying whether the workers reached the goal set by the organization.

Previous research holds significance in identifying the factors influencing job performance, providing ideas on desirable behaviors and attitudes for job performance, and suggesting effective measures for organizational effectiveness. In response, this study regards job performance as various business activities performed by workers to accomplish the organizational goals and behaviors achieved by the continuous efforts of the workers.

### 2.5. Support for Career Development Intelligence Building and Job Performance

Support for career development intelligence building makes workers show as many personal competences as possible while contributing to both individual and organizational performance [45]. As suggested in the research by Lee and Cho [46], the workers in the organization with well-designed support for career development intelligence building environment for employees commit to their roles and actively participate in all organizational activities, including both job- and performance-related activities.

Numerous studies support the work of Rich et al. Skinner, Wellborn, and Connell [47] pointed out that support for career development intelligence building enhances job performance while lowering turnover intention. Singh and Jha [48] also explained that hotel workers’ education has a statistically positive influence on job performance [49,50].

While various studies have reported a positive relationship between education and job performance, there are only a few studies on the mechanism of this direct relationship. Thus, to address this issue, Lim et al [51] studied the importance of proving the influencing relationship between variables such as job model, support for career development intelligence building, self-esteem, and job performance. They said that only a few studies had covered their relationship at the general level [52]. In response, Seo, Jang, and Kim [53] attempted to clarify this relationship by focusing on the effects of support for career development intelligence building as the variable of positive attitude changes instead of negative burnout in the job demands-resources model. Their work revealed that job engagement enhances self-efficacy and job performance and that self-efficacy has a direct positive influence on job performance.

Like self-efficacy, self-esteem is an individual’s subjective evaluation of their worth. Workers with positive self-esteem show higher job satisfaction, which is positively related to product and service factors and competitiveness factors that differ depending on individual efforts rather than financial performance, such as sales that individuals cannot control [54,55]. Notably, research on resort workers shows that self-esteem plays a mediating role in the relationship between resort workers’ emotional disharmony and job satisfaction [56,57]. In the same vein, self-esteem is expected to mediate the relationship between psychological variables and other variables related to organizational effectiveness.

In the case of learning transfer, it had a mediating effect on the influencing relationship between organizational learning and organizational performance [58]. The relationship between program satisfaction, job satisfaction, and career commitment [59,60] and the influencing relationship between learners’ characteristics, job satisfaction, and organizational commitment [61]. In particular, learning transfer played a mediating role in the variable associated with organizational effectiveness. Therefore, it can be assumed that learning transfer plays a mediating role between job engagement and job performance in reflecting an individual’s psychological characteristics.

In summary, support for career development intelligence building has a positive influence on job performance, whereas self-esteem and learning transfer play a mediating role in the relationship between support for career development intelligence building and job performance. This understanding will help identify the relationship between an individual’s psychological variables that are important for predicting job performance.

### 2.6. The Mediating Role of Learning Transfer and Self-Esteem between Support for Career Development Intelligence Building and Job Performance

Regardless of how motivated learners are to participate in learning, they are inclined to continue their participation selectively depending on their self-esteem [62]. Accordingly, this confidence in one’s worth and abilities is a vital element in examining successful careers [58] and continuity of career development [35]. Learning transfer as a concept has also been used for evaluating individuals’ perception of their own jobs, and in reference [57], the author defined learning transfer as either a positive or negative attitude toward job performance. Conceptualization of one’s learning may not only relate to job performance but also influence other behavioral types among newly hired workers, according to whom, learning transfer is a variable that affects job performance or adjustment at work [60]. Interestingly, it was found that students with high learning transfer have high levels of adjustment in their job performances.

Support for career development intelligence building through training and development affects their adaptation to career life, such as relationships with peers and bosses and their attitudes toward work, in varying degrees [63]. Learning transfer is created through social interactions involving recognition, respect, acceptance, and evaluation of values imparted by significant others and successful individual experiences [61].

According to the career motivation model proposed by London and Noe [64], individual career elasticity occurs due to situational factors affecting individuals, which ultimately induces changes in individual behavior by immersing them in career competency. In summary, career development intelligence building support helps organizational members set and review their future career paths on their own, and at the organizational level, it helps to systematically analyze the growth and development processes of organizational members that meet the needs of the organization [65]. In this respect, it can be inferred that the career development intelligence building support provided to organizational members strengthens their career-related capabilities, which leads to actions for individual career development and Job performance improvement. In fact, various previous studies have suggested that career development support provided by organizations positively affects the growth of organizational members and the performance of the entire organization [66,67]. Based on this, a number of recent companies have provided various career development support programs for corporate workers based on the importance of effective human resource development in a rapidly changing business environment [68].

## 3. Research Methodology

### 3.1. Research Model

**Figure 1:**
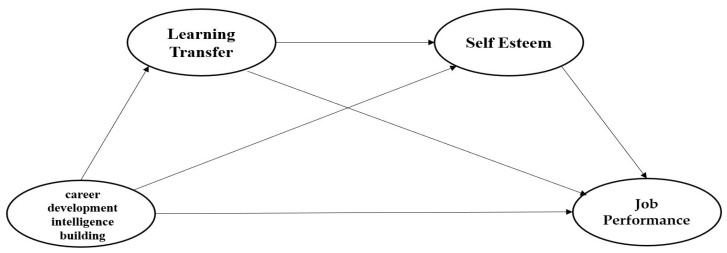
Research Model 1.

The objective of this study is to determine whether learning transfer and self-esteem have a mediating effect on the relationship between support for career development intelligence building and job performance among newly hired Korean workers. To this end, a research model was constructed, as shown in Figure 1.

### 3.2. Participants

This study was undertaken using 18,081 data points obtained from the 2018 Graduate Occupational Mobility Survey (GOMS) panel data by the Korea Employment Information Service, which were the most recently collected and organized. GOMS panel data was designed to provide the data for building infrastructure for manpower supplies and demands forecasting in Korea and was funded by Korea Employment Insurance organization. This panel survey was reviewed by the Korea Employment Information Service Institute Institutional Review Board (IRB) and executed using the tablet assisted personal interview (TAPI) method for multi-stage stratified sampled newly hired workers leading by Korea Research agency.

Among the 18,081 participants, 9769 were men (54.0%) and 8312 were women (46.0%), and the number of men was slightly higher than that of women. The average age was 26 years (SD = 4.661), and 16,326 participants (90.3%) were in their 20s. Lastly, in terms of residence, the highest number of participants lived in Seoul (5023 subjects, 27.8%), followed by Gyeonggi-do (3685 subjects, 20.4%) and Busan (1302 subjects, 7.2%) (Table 1).

### 3.3. Measures

To measure support for career development intelligence building, the questionnaire within this panel data consisted of seven questions regarding overall satisfaction with the workplace (work details, work environment, personal development possibility, work-related education, or training), which were measured on a scale of 5 points from “very dissatisfied” to “very satisfied.” Learning transfer is a question that has been used in a number of related studies [1,16] and is divided into two parts: satisfaction with education and use of training. Self-esteem was measured following Kim’s [6] study. Accordingly, self-esteem was regarded as a part corresponding to the respondents’ personal, relational, and situational satisfaction with individual mental health; the higher the scale, the higher the self-esteem. The job performance survey consists of subjective job performance levels and job performance satisfaction, developed by the Korea Employment Information Service. GOMS Panel survey questionnaires were approved by the National Statistical Office (Statistics Office approval number: 327004).

### 3.4. Data Analysis and Research Procedures

This study used SPSS21.0, AMOS22.0, and PROCESS macro for analysis. The analysis procedure was structured as follows. First, we conducted a frequency analysis of the independent, dependent, and mediating variables. In addition, Cronbach’s α analysis of variables was conducted to confirm the variables’ reliability level. Second, a confirmatory factor analysis of the variables was used to identify the goodness of fit. Through this step, the validity of the research model was confirmed. Third, we analyzed the correlation between the variables and the direction of their relationship. Fourth, we set the full and partial mediation models and adopted the appropriate model for the research. In this step, the model fit and χ2 of the two models were compared to determine which model is more suitable for the study. Fifth, the covariance structure was analyzed based on the structural equation along with the model’s goodness of fit and the effects of the variables. In this step, the relationship between the variables was confirmed. Finally, we analyzed the mediating effect using the PROCESS macro, an analysis method that simultaneously tests the direct, mediating, and moderating effects without a separate process [9]. According to the PROCESS macro can test the mediating effect using bootstrap. In this step, the mediating effect was confirmed through process macro analysis.

## 4. Research Results

This study first analyzed the confirmatory factors to test the research model’s goodness of fit. For higher parsimony, this study parceled the data on education, training, and job performance and randomly set three sub-variables. In terms of self-esteem and job performance, we set three variables for self-esteem and two sub-variables for job performance based on theoretical background analysis.

Subsequently, the study examined whether each variable was set appropriately and checked the model’s goodness of fit based on the goodness-of-fit index, as shown in Table 2.

For the next step, we analyzed the correlations among variables as shown by the means, standard deviations, Cronbach’s α, and correlations between the study variables in Table 3. All Cronbach’s values were over 0.6, and all variables in the model had a direct relationship with each other. The correlation analysis confirmed that there was statistical significance in support for career development, intelligence building, learning transfer, self-esteem, and job performance.

A partial mediation model was constructed in relation to the research question. The fit indices of the partial mediation model are presented in Table 4. The partial mediation model showed significance: χ^2^ = 1090.640 (df = 45, *p* < 0.001), NFI = 0.903, CFI = 0.845, and RMSEA = 0.090. The arrow from support for career development intelligence building to job performance was deleted to create an alternative full mediation model. The index of fit of the full mediation model was significant (χ^2^ = 1088.530, df = 44, *p* < 0.001), with NFI = 0.900, CFI = 0.900, and RMSEA = 0.093. As shown in Table 4, compared with the partial mediation model, the full mediation model showed a slight difference, with a one-point difference in fit level.

**Figure 2:**
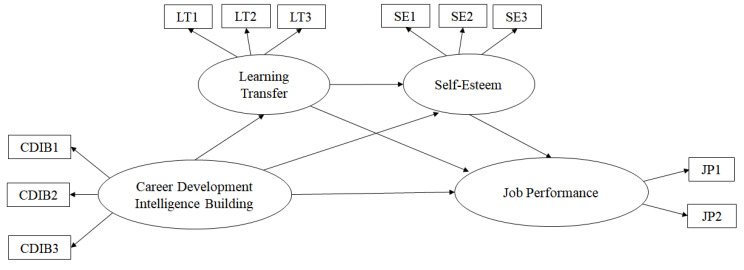
Partial mediation model.

**Figure 3:**
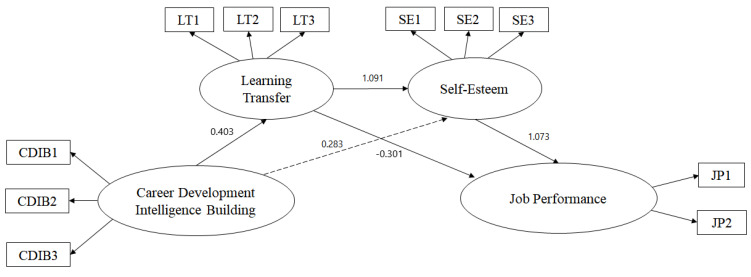
Full mediation model.

Next, a model comparison was conducted with the use of the difference in values between the two models to compare partial and full mediation models. According to the results, the difference in χ^2^ was 2.11, smaller than the threshold of 3.84, with a difference of df 1. It shows that the difference in χ^2^ was not significant. Model verification did not reveal significant results when comparing the values and degrees of freedom of the two models. In this case, a model with a higher degree of freedom is likely to be selected (Kelloway, 2016). The full mediation model with a higher degree of freedom was selected for a comparison between the full and partial mediation models. In other words, the full mediation model was determined as more appropriate than the partial mediation one through a comparison of the goodness of fit and the difference in χ^2^. Figure 2 presents a partial mediation model, which is a full mediation model; Figure 3 presents a full mediation model, which is a partial mediation model.

A structural equation was created to answer the research questions, and a covariance structural analysis between the study variables was conducted. The standardized route (β), standard error (S.E), and t- and *p*-values were reviewed in the selected full mediation model to analyze the relationships between the major variables. The results of the analysis are presented in Table 5. Support for intelligence building in career development had a significant positive relationship with self-esteem (β = 0.403, *p* < 0.001). The relationship between support for intelligence building in career development and self-esteem was significant (β = 1.091, *p* < 0.001). The relationship between learning transfer and self-esteem was significantly positive (β = 0.0283, *p* < 0.001). Accordingly, the relationship between self-esteem and job performance was also significantly positive (β = 1.073, *p* < 0.001).

Lastly, this study used Model 6 (Hayes, 2013) of PROCESS. The results are shown in Table 6. The indirect effect between support for career development intelligence building and parameters, including learning transfer, self-esteem, and job performance, was 0.3189. As the 95% confidence interval did not include 0, this showed the significance of the mediating effect.

## 5. Discussion

This study aimed to identify the influence of support for career development intelligence building for college graduates on their job performance among 18,081 newly hired workers in Korea, using GOMS 2018 panel data, and to verify the mediating effect of learning transfer and self-esteem in the relationship between career development intelligence building and job performance.

According to the results of research, career development intelligence building for college graduates turned out to represent a significantly positive relationship in their job performance through learning transfer and self-esteem. In addition, through this study, it was confirmed that career development intelligence building ended up indirectly influencing job performance through learning transfer and self-esteem instead of directly influencing it. Discussion and conclusion are as follows, based on results of the study.

First, these results differed from Yang et al.’s [67] findings, which showed that learning transfer was directly associated with job performance and correlated with job self-efficacy among college graduates. In the present study, learning transfer had no direct association with job performance; however, the higher the level of learning transfer among college graduates, the more likely it was for them to positively evaluate themselves, which strengthened high levels of learning transfer.

Second, learning transfer and self-esteem mediated the relation between support for career development intelligence buildings and job performance. Taking into consideration that the value between the 95% confidence intervals did not contain 0 in the results of this study, the mediating effect showed a statistical significance. Moreover, the indirect effect between the independent variable, career development intelligence building; the parameters learning transfer and self-esteem; and the dependent variable, job performance was found to be 0.3189.

According to the former studies [42], the higher the level of learning transfer, the more likely it was for college graduates to enhance their motivation and performance, although they could not identify any mediating variables. Hence, further research is required to clarify the effects of various variables vis-à-vis support for intelligence building in career development.

Nevertheless, the study results support the need to develop specific programs to improve learning transfer and self-esteem, as these variables have a close relation with job performance among college graduates. From this perspective, it is essential to determine how support for career development intelligence building can be promoted and to help college graduates improve their learning transfer and self-esteem through counseling with suitably qualified career advice to ensure they advance successfully to college and are able to face up to experiences such as failure, adversity, and psychological pain, and remain focused on job performance.

The followings based on the findings of this study are presented to enhance the job performance of college graduates. First, it is necessary to supplement and realistically increase support for career development. Second, it is required to reinforce career development programs and organization-friendly systems. A limited time for bosses and employees to spend together may result in fewer opportunities for them to interact with each other in a positive way. Third, initiating and diversifying programs is needed to increase learning transfer among new employees. Learning transfer has been shown to have positive influence on job performance and has a significant relation with college graduates’ subsequent lives. Thus, institutional support is required for development and revitalization of various activities to increase students’ learning transfer. In other words, learning transfer improvement programs are required to allow participation by organizations.

This study has significance in that it demonstrated the influence of support for career development intelligence building on college graduates’ job performance and the fact that self-esteem and learning transfer are mediating factors. The limitations that this study has are as follows. First, the participants were all college graduates. Further studies should address experienced or high school graduates as subjects to determine the generalizability of this study’s findings. Second, this study recognized that organizations are an important environmental system that provides college graduates with services for their growth and development. Third, the questionnaire to measure network variables was not sophisticated, which made it difficult to measure multiple questions grouped together; that is, there was a limitation in accurately measuring the responses to the questionnaire inquiring about support for career development, intelligence building, and self-esteem, among others. To be specific, it did not reflect the respondents’ propensity to pursue both functions since they had to choose one of the two questionnaires to measure their job performance. Hopefully, further research will elaborate on the variables associated with the study according to its purpose.

## Figures and Tables

**Table 1:** Participants.

Categories		Number (Percentage %)	Note
Sex	Male	9769 (54.0)	
	Female	8312 (46.0)	
Age	20s	16,326 (90.3)	M = 26.742 SD = 4.661
	30s	1123 (6.2)	
	Missing Value	632 (3.5)	
Residential Area In Korea	Seoul	5023 (27.8)	
	Pusan	1302 (7.2)	
	Daegu	902 (5.0)	
	Daejeon	721 (4.0)	
	Incheon	825 (4.6)	
	Gangju	602 (3.3)	
	Ulsan	407 (2.3)	
	Kyunggi	3685 (20.4)	
	Kangwon	545 (3.0)	
	Cheongbuk	488 (2.7)	
	Cheongnam	464 (2.6)	
	Jeonbuk	610 (3.4)	
	Jeonnam	422 (2.3)	
	Kyungbuk	743 (4.1)	
	Kyungnam	1093 (6.0)	
	Jeju	126 (0.7)	
	Sejong	123 (0.7)	

**Table 2:** Goodness-of-fit Indexes for Structural Equation Model.

χ2	df	CFI	NFI	RMSEA	*p*
1090.640	45	0.902	0.903	0.090	0.000

**Table 3:** Number, Means, Standard Deviations, Cronbach’s α, Correlations, and Reliabilities of Study Variables.

Variable	Mean	SD	Cronbach’s α	1	2	3	4
1. Support for career development intelligence building	3.789	0.495	0.772	-			
2. Learning Transfer	3.369	0.447	0.701	0.485 *	-		
3. Self-Esteem	3.389	0.501	0.698	0.471 *	0.428 *	-	
4. Job Performance	3.752	0.875	0.801	0.249 *	0.268 *	0.388 *	-

* *p* < 0.001.

**Table 4:** Goodness-of-fit Indexes for the Structural Equation Model.

Model	χ^2^	df	CFI	NFI	RMSEA	*p*
Partial Mediation Model	1090.640	45	0.902	0.903	0.090	0.000
Full Mediation Model	1088.530	44	0.900	0.900	0.093	0.000

**Table 5:** The path estimates of full mediation model.

Path	Path Coefficient	Standard Error	C.R.	*p*
Support for career development intelligence building → Learning Transfer	0.403	0.029	24.345	0.000
Learning Transfer → Self-Esteem	1.091	0.240	8.879	0.000
Support for career development intelligence building → Self-Esteem	0.0283	0.044	0.449	0.498
Self-Esteem → Job Performance	1.073	0.087	11.995	0.000
Learning Transfer → Job Performance	−0.301	0.089	−3.349	0.035

**Table 6:** PROCESS Macro: Analysis Results.

Path	Indirect Effect	LLCI	ULCI
Support for career development intelligence building → Learning Transfer → Self-Esteem → Job Performance	0.3189	0.1915	0.3124

## Data Availability

The data presented in this study are available in article.
